# Will Wind Turbines Affect the Distribution of Alashan Ground Squirrel? Insights from Large-Scale Wind Farms in China

**DOI:** 10.3390/biology14070886

**Published:** 2025-07-19

**Authors:** Yuan Wang, Wenbin Yang, Qin Li, Min Zhao, Ying Yang, Xiangfeng Shi, Dazhi Zhang, Guijun Yang

**Affiliations:** 1 School of Life Science, Ningxia University, Yinchuan 750021, China; oliver-wy@foxmail.com (Y.W.);; 2 Institute of Design and Agricultural Survey in Ningxia, Yinchuan 750002, China

**Keywords:** *Spermophilus alashanicus*, spatial distribution, burrows, environmental factors, wind farm, semi-desert grassland

## Abstract

The wind energy resources in the northwestern desert and semi-desert grassland regions of China are abundant. However, the ramifications of large-scale centralized wind farm operations on terrestrial rodents remain incompletely understood. In May and September 2024, we employed a grid sampling method combined with burrow counting and kernel density analysis to investigate the spatial distribution of Alashan ground squirrel (*Spermophilus alashanicus*) burrows in different wind turbine power zones (control, 750 kW, 1500 kW, 2000 kW, and 2500 kW) at the Taiyangshan wind farm in China. Using generalized additive models and structural equation models, we analysed the relationship between burrow spatial distribution and environmental factors. The results revealed no significant linear correlation between burrow density and turbine layout density, but was significantly positively correlated with turbine power (*p* < 0.05). The highest burrow density was observed in the 2500 kW zone, with values of 24.43 ± 7.18 burrows/hm^2^ in May and 21.29 ± 3.38 burrows/hm^2^ in September (*p* < 0.05). The squirrels exhibited a tendency to avoid constructing burrows within the rotor sweeping areas of the turbines. The burrow density distribution exhibited a multinuclear clustering pattern in both May and September, with a northwest–southeast spatial orientation. Turbine power, aspect, and plan convexity had significant positive effects on burrow density, whereas vegetation height had a significant negative effect. Moreover, vegetation height indirectly influenced burrow density through its interactions with turbine power and relief degree. Under the combined influence of turbine power, topography, and vegetation, Alashan ground squirrels preferred habitats in low-density, high-power turbine zones with shorter vegetation, sunny slopes, convex landforms, and minimal disturbance.

## Simple Summary

Wind energy is widely promoted as a clean energy source, but its ecological impacts, especially in grassland ecosystems, are not fully understood. This study looked at how wind farms in the desert steppe of northwestern China affect the burrowing behaviour of the Alashan ground squirrel (*Spermophilus alashanicus*), a typical ground-dwelling rodent. We found that these animals tend to avoid building burrows directly under wind turbines, especially within the area swept by the blades. Interestingly, the number of effective burrows increased in areas with higher turbine power. Burrow locations were also influenced by terrain and vegetation height. These findings help us better understand how wind farms may influence local wildlife and provide useful information for balancing renewable energy development with grassland conservation.

## 1. Introduction

Wind energy is increasingly recognized as a vital sustainable energy source, particularly due to global warming, which drives many countries to promote renewable energy generation. Various policies have been implemented globally to promote the development of wind energy [1]. The European Union has set ambitious targets for renewable energy, aiming for at least 42.5% of its energy consumption to come from renewable sources, and with an aspirational goal of 45% by 2030 [2]. Similarly, countries like China and the United States have introduced incentives and subsidies to encourage investment in wind power projects [3,4]. According to the Global Wind Energy Council, the expected average annual installation is 93.3 GW during 2022–2026, with an additional 466 GW likely to be installed in total [1]. The growth of wind power capacity globally highlights its essential role in reducing greenhouse gas emissions and addressing energy security issues [5].

Despite their benefits, the environmental and ecological impacts of wind farms have faced increased scrutiny. Since the 1990s, there have been numerous reports detailing the negative effects of wind energy development on the environment and wildlife [6]. Among the affected wildlife, flying animals, particularly birds and bats, have received significant attention due to the risk of mortality from collisions with wind turbine blades. Studies have shown that wind turbines pose a substantial threat to avian populations, with estimates suggesting that hundreds and thousands of birds are killed annually due to collisions with turbine blades [7]. Operating wind farms raises the risk of bird collisions [8], causing habitat loss and fragmentation [9], and altered migratory routes. Additionally, noise from rotating wind turbines disrupts bats’ echolocation, while changes in atmospheric pressure may impair their respiratory functions [10]. These challenges not only contribute to a significant loss of biodiversity, but may also disrupt ecological balances, with potential long-term consequences [11].

In addition to directly affecting wild animals, wind farms will also change the natural environment that organisms rely on for survival. Wind farm operations can raise nighttime surface temperatures, reduce the net primary productivity of vegetation [12,13], and negatively affect soil properties [14]. The construction and operation of wind farms can alter the territorial ranges of animals [15,16,17,18,19,20] and influence their breeding and foraging behaviours [21,22,23]. The abundance of earthworms in soil within 200 m of wind turbines is significantly affected by the low-frequency noise produced during turbine operation [24]. The antipredator behaviour of the California ground squirrel (*Otospermophilus beecheyi*) is altered by exposure to turbine noise [25]. Terrestrial vertebrates may experience habitat disruption and fragmentation due to the installation of wind farms, which can lead to altered movement patterns and decreased population viability [26]. However, the disturbances caused by wind farm operations vary in their effects on different terrestrial animal species. The European hamster (*Cricetus cricetus*) does not exhibit avoidance behaviour in response to wind turbines [27]. In agricultural landscapes, the common vole (*Microtus arvalis*) and the striped field mouse (*Apodemus agrarius*) exhibit different physiological stress responses to wind turbine operation [22]. Furthermore, no significant differences were observed in the community characteristics of *Apodemus agrarius* and *Microtus arvalis* between turbine sites and control areas in mountainous wind farm landscapes [28].

Compared to other biological groups, terrestrial animals living near wind farms have received relatively little attention [22,24,28]. The Alashan ground squirrel (*Spermophilus alashanicus*), belonging to the order Rodentia and the family Sciuridae, primarily inhabits desert grassland and semi-desert ecosystems within western Inner Mongolia, Ningxia, and northern Gansu in China. This social species exhibits hibernation behaviour and prefers to build its burrows in elevated, dry, open grasslands that have a specific slope gradient, which constitutes its ideal habitat. The dietary habits of these squirrels involve consuming the tender shoots and seeds of plants, resulting in localized damage around their burrows and contributing to grassland degradation [29]. Its excavation activities often lead to extensive barren areas and soil erosion. Many predators rely on rodents as a food source. The Alashan ground squirrel in the wind farm region could attract predatory birds, elevating the risk of bird strikes, which may have adverse effects on both ecological balance and sustainable development. Analysing the distribution of rodents is vital for assessing the environmental impact of wind farms.

The Ningxia Hui Autonomous Region has rich wind energy resources, especially in its desert and semi-desert grasslands, creating favourable conditions for developing large wind farms. The Taiyangshan wind farm, established in 2008, is a large-scale facility located in the semi-desert region of central Ningxia, home to a significant population of Alashan ground squirrels. This study investigated the distribution of Alashan ground squirrel burrows within the wind farm, aiming to address the following questions: (1) Is there a correlation between the distribution of burrows and the deployment density and capacity of different wind turbines? (2) What are the key environmental factors influencing the density of burrows in the wind farm area? The findings aim to understand the spatial distribution patterns of Alashan ground squirrels in wind farm environments, providing valuable insights for the operational management and environmental impact assessments of wind farms.

## 2. Materials and Methods

### 2.1. Study Area

The Taiyangshan wind farm is situated at the intersection of Lingwu City, Litong District, Hongsipu District, and Yanchi County in central Ningxia. It was established in 2008, located at the edge of the Ordos Plateau. The terrain is characterized by low mountains and hilly landscapes, with undulating but relatively open topography. Most slopes are gentle, and the elevation ranges from 1176 to 1446 m, gradually increasing from the northwest to the southeast. The average elevation is approximately 1300 m, with a maximum relative elevation difference of around 270 m. The annual average temperature in this region is 9 °C, with an extreme maximum of 37.4 °C and an extreme minimum of -27.1 °C. Additionally, the average annual precipitation is 266.1 mm, and the average wind speed is 3.2 m/s. The vegetation includes desert and semi-desert grasslands, with a few small crescent-shaped dunes scattered throughout the area. The main vegetation in the study area includes *Suaeda prostrata*, *Suaeda glauca*, *Lespedeza potaninii*, *Artemisia scoparia*, *Convolvulus tragacanthoides*, and *Caragana korshinskii*, among others.

### 2.2. Sample Methods

**Figure 1:**
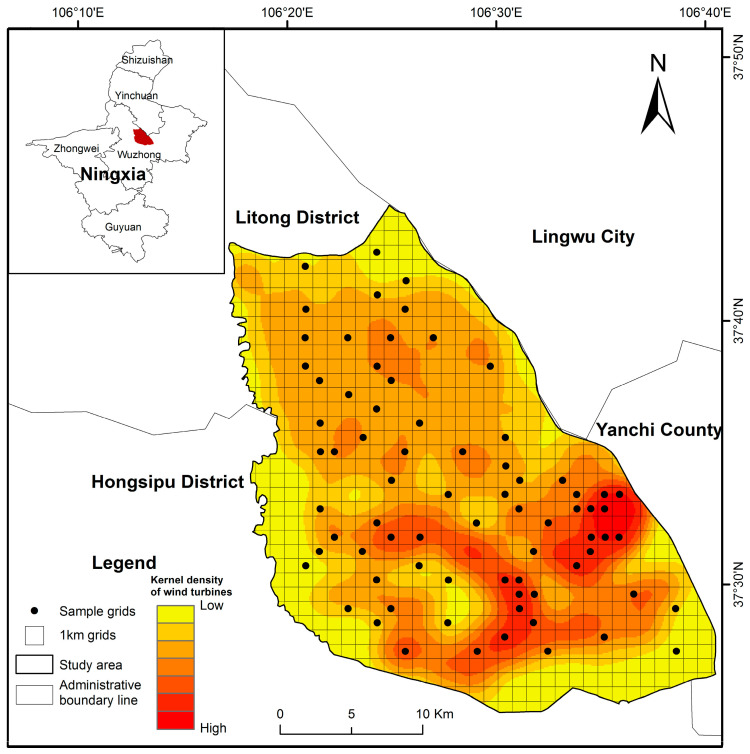
Research area and sample grids of Alashan ground squirrel on the Taiyangshan wind farm.

The study area is located at 106°17′4′′~106°39′58′′ E and 37°25′32′′~37°28′3′′ N, covering approximately 730 km^2^. Based on the technical guidelines for turbine density in onshore wind farms [30], using the grid analysis method (Figure 1), we divided the study area into 1 km × 1 km grids using ArcGIS 10.8 [31]. The total number of grids obtained was 812 (including incomplete grids located along the edges of the study area). The number of wind turbines per grid ranged from 0 to 11. A total of 877 turbines were recorded, resulting in an average turbine density of 3.16 units/km^2^. Areas of higher turbine density were predominantly concentrated in the southeastern sector of the study area. To avoid interference from other potential environmental factors that may influence the distribution of Alashan ground squirrels—and thereby confound the relationship between squirrel presence and wind energy development—we excluded grids that contained villages, highways, photovoltaic panels, or livestock farms. Additionally, grids located at the edges of the study area were removed to prevent edge effects. The power capacity of wind turbines varied among different grids. To investigate the distribution of Alashan ground squirrels under different wind turbine characteristics, the remaining 566 grids were classified into five groups based on the power capacity of the wind turbines within each grid: 0 kW (control group), 750 kW, 1500 kW, 2000 kW, and 2500 kW. Using a stratified random sampling method [32], we selected at least 10% of the grids from each turbine capacity category, resulting in a total of 75 grids as survey samples. These grids covered all types of wind turbine installations and provided strong representativeness, allowing for a comprehensive reflection of the overall conditions in the Taiyangshan wind farm, which allowed us to compare the distribution patterns of Alashan ground squirrels across different turbine capacity categories (Table 1). We defined the grids without wind turbines as the control group. A 100 m × 100 m sample plot was established in the centre of each grid.

### 2.3. Burrow Survey

Based on both literature and field observations [29,33,34], the Alashan ground squirrel typically occupies a single burrow per individual—except during the breeding season. Each burrow generally has a single entrance, making burrows a suitable proxy for estimating population density. Active burrows were identified by smooth, intact entrances free of cobwebs, often accompanied by nearby footprints, fresh soil, and occasionally faecal or urinary traces. In contrast, abandoned burrows were characterized by entrances blocked by vegetation or covered with cobwebs, and lacked visible footprints nearby. A 100 m × 100 m sample plot was established at the centre of each survey grid cell. Five investigators walked in parallel transects, each surveying a belt approximately 20 m wide. When an active burrow was encountered, its geographic coordinates were recorded using a GPS device (Zhuolin Electronic Technology Co., Ltd., Wanguo Building, Yaohai District, Hefei, China, model: A6), and a small, brightly coloured flag was placed nearby to mark the location and prevent duplicate recordings by other investigators. After completing the field survey, the burrow data from each sampling point were compiled in the lab, and the density of effective burrows (DEB) per hectare (hm^2^) was then calculated. Taking advantage of the species’ hibernation cycle, Alashan ground squirrel populations were surveyed twice: during the post-hibernation emergence period in late spring (5–14 May) and prior to the onset of autumn hibernation (18–26 September), as previously documented [29].

### 2.4. Environmental Factors

Four major categories of environmental variables were selected for further analysis based on their potential influence on the distribution of the Alashan ground squirrel: wind power-associated, climatic, vegetative, and topographic factors.

Wind power-associated factors comprised the number of wind turbines within grid cells, wind turbine power (kW), tower height (m), wind turbine rotor diameter (m), and the minimum distance (m) from the sample plot centroid to the nearest turbine.

The climate factors were the maximum temperature, minimum temperature, and mean precipitation, sourced from the Worldclim global climate data platform (https://www.worldclim.org/) (accessed on 13 February 2025) with a resolution of 30 arc seconds.

Vegetation factors were collected using standardized sampling methods. The five-point sampling method was used to set 5 m × 5 m plots (for shrubs) or 1 m × 1 m plots (for herbs) in the survey sample plot, and the survey measured flora diversity, flora abundance (per m^2^), flora richness, flora height (cm), and soil-adjusted vegetation index (SAVI) from the Geographic Condition Monitoring Cloud Platform (http://www.dsac.cn) (accessed on 21 February 2025) with a spatial resolution of 30 m × 30 m.

Topographic factors, including transformation of aspect, slope, plan curvature, profile curvature, and relief degree, were extracted from the regional Digital Elevation Model (DEM) using ArcGIS 10.8.

Admittedly, predation pressure from potential predators may also influence the distribution of Alashan ground squirrels. As this study focused on the potential relationship between wind energy development and squirrel distribution, we did not quantify the effects of predators.

### 2.5. Data Analysis

#### 2.5.1. Kernel Density Estimation

Kernel density estimation was used to assess the spatial density of point features across the study area, providing insight into their degree of spatial aggregation. Its calculation formula is

(1) $$f\left(x\right)=\frac{1}{nh}\sum_{i=1}^{n}k\left(\frac{x-x_{i}}{h}\right)$$

In this formula, *f*(*x*) denotes the estimated kernel density value; $k(\frac{x-x_{i}}{h})$ represents the weight function; $\left(x-x_{i}\right)$ indicates the distance from the evaluation point *x* to the sample *x_i_*; *h* signifies the preset search radius; and *n* indicates the number of point features.

#### 2.5.2. Spatial Autocorrelation

We employed the global Moran’s *I* to assess the spatial autocorrelation of the DEB and standardized *Z*-values to evaluate its statistical significance [35]. Moran’s *I* ranges from −1 to 1. If Moran’s *I* > 0 (*p* < 0.05), it indicates a positive spatial correlation, meaning that high (or low) DEB values are significantly clustered together. If Moran’s *I* equals 0 or is close to 0, it suggests no spatial autocorrelation between adjacent regions, indicating a random distribution of the DEB. Conversely, if Moran’s *I* < 0 (*p* < 0.05), it signifies a negative spatial correlation, implying that DEB values are spatially dissimilar or exhibit opposite characteristics. The global Moran’s *I* is considered significant at *p* < 0.05 and |*Z*| > 1.96. The calculation formula is as follows:

(2) $$I=n\sum_{i=1}^{n}\sum_{j=1}^{m}\frac{W_{ij}\left(x_{i}-\bar{x}\right)\left(x_{j}-\bar{x}\right)}{\sum_{i=1}^{n}\sum_{j=1}^{m}W_{ij}{\left(x_{i}-\bar{x}\right)}^{2}}$$

In the formula, *n* denotes the total number of research units, while *x_i_* and *x_j_* represent the DEB in the *i*th and *j*th study areas, respectively. The spatial weight coefficient, *W_ij_*, reflects the spatial relationship between the *i*th and *j*th regions; it equals 1 if the regions are adjacent and 0 otherwise.

#### 2.5.3. Standard Deviation Ellipse Analysis

By quantitatively analysing the spatial distribution range and essential parameters—such as the centre, major axis, minor axis, and azimuth angle of the standard deviation ellipse—we can gain a clearer understanding of the DEB distribution’s centrality, directionality, and spatial morphology in the study area.

#### 2.5.4. Generalized Additive Model and Structural Equation Model

We employed the Spearman correlation coefficient to analyse the correlation between DEB and various environmental factors. We utilized the variance inflation factor (VIF) to evaluate for collinearity among the explanatory variables. Additionally, we used the Generalized Additive Model (GAM) to analyse how environmental factors affect the distribution of the DEB [36]. The calculation formula is as follows:

(3) $$g\left(\mu \right)=\alpha +f_{1}\left(x_{1}\right)+f_{2}\left(x_{2}\right)+\ldots +f_{n}\left(x_{n}\right)$$

In the equation, *f*_1_*(x*_1_*), f*_2_*(x*_2_*), …, f_n_(x_n_)* denote the smoothing functions for the explanatory variables. We used the Akaike information criterion (AIC) to select the optimal influencing factors through stepwise regression. The coefficient *R*^2^ was used to determine the goodness of fit of the model, and the *p*-value was used to evaluate the significance of each influencing factor.

A path analysis was developed using partial least squares structural equation modelling (PLS-SEM) to evaluate the impact of key influencing factors on changes in burrow density.

This study used Origin 2021 for the grouped statistical analysis of DEB data, ArcGIS 10.8 for kernel density distribution, spatial autocorrelation analysis, and mapping, and S-PLUS 8.0 for constructing and visualizing the GAM. PLS-SEM was performed using SmartPLS 4.0.

## 3. Results

### 3.1. Variation of Alashan Ground Squirrel’s Burrow Density

#### 3.1.1. DEB Distribution in Different Wind Turbine Density Areas

**Figure 2:**
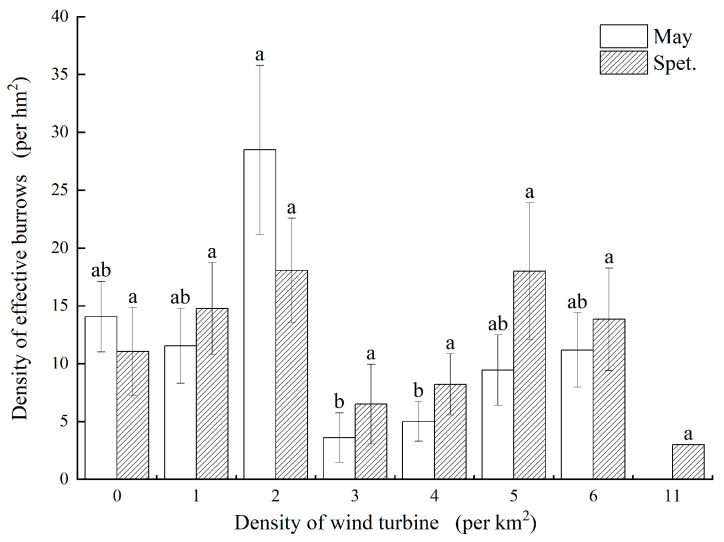
Comparison of the DEB in different wind turbine density areas. Different small letters mean a significant difference at the 0.05 level.

The analysis of DEB changes in grid cells with different wind turbine densities showed that during the survey periods in May and September (Figure 2), the grid cells with a wind turbine density of 2 turbines/km^2^ had the highest DEB, reaching 28.50 ± 7.30 burrows/hm^2^ and 18.21 ± 4.41 burrows/hm^2^, respectively. In contrast, grid cells with a wind turbine density of 11 turbines/km^2^ exhibited the lowest average DEB in both May and September.

#### 3.1.2. DEB Distribution in Different Wind Turbine Power Areas

**Figure 3:**
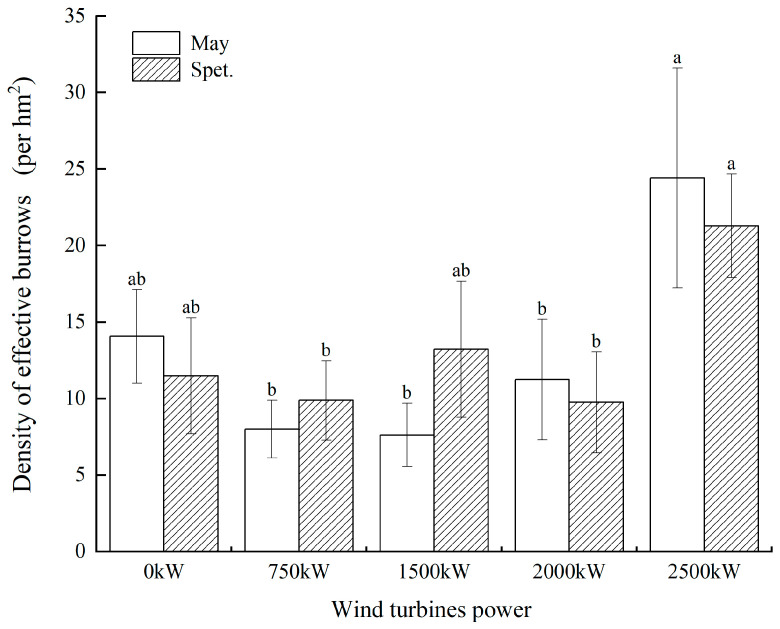
Comparison of the DEB in different wind turbine powers areas. Different small letters mean a significant difference at the 0.05 level.

The survey results of the DEB in areas with different wind turbine powers (Figure 3) showed that the DEB in the 2500 kW wind turbine area was significantly higher than that in other power areas in May (24.43 ± 7.18 burrows/hm^2^) and September (21.29 ± 3.38 burrows/hm^2^) (*p* < 0.05). The area with the lowest average DEB in May was the 1500 kW wind turbine area, while that in September was the 2000 kW wind turbine area. The average DEB in the control area was at a medium level.

**Figure 4:**
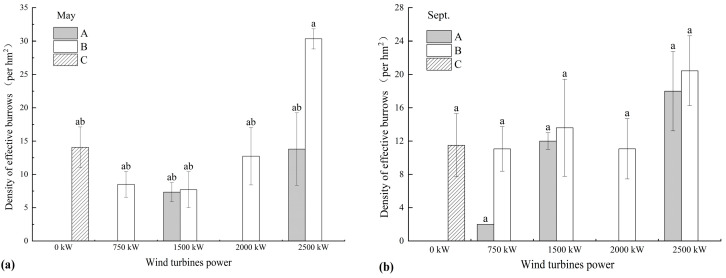
Density of effective burrows (DEB) in different distance zones from wind turbines with varying power levels in (**a**) May and (**b**) September. Zone A: within the rotor diameter range; Zone B: from the rotor diameter to 500 m; Zone C: beyond 500 m. Different lowercase letters indicate significant differences at the 0.05 level.

Given the presence of wind turbines with varying power capacities in the study area, and the established correlation between noise emissions and rotor diameter, where turbine noise typically becomes indistinguishable from ambient noise beyond 500 m, this study classified the distance to the nearest turbine into three zones (Figure 4): Zone A (within the rotor diameter of the turbine), Zone B (from the rotor diameter to 500 m), and Zone C (beyond 500 m). The rotor diameters corresponding to 750 kW, 1500 kW, 2000 kW, and 2500 kW turbines are 50 m, 100 m, 120 m, and 150 m, respectively. Analysis of DEB variations at different distances from the nearest turbine across areas with different turbine power levels showed that the DEB was consistently lower within the rotor diameter range (Zone A) than in the intermediate range (Zone B). Additionally, no effective burrows were observed within the rotor diameter zone of the 750 kW turbine area in May or within the rotor diameter zone of the 2000 kW turbine area in both May and September.

### 3.2. Spatial Distribution Characteristics of DEB

**Figure 5:**
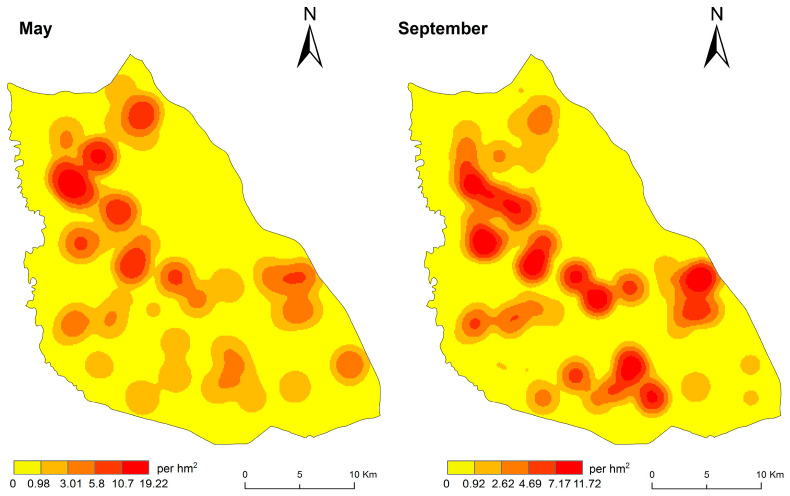
Kernel density distribution of the DEB in the Taiyangshan wind farm in May and September.

The natural breaks classification method was applied to visualize the spatial distribution of the DEB in the Taiyangshan wind farm (Figure 5). In May (Moran’s *I* = 0.151, *p* = 0.006, *Z* = 2.647) and September (Moran’s *I* = 0.133, *p* = 0.02, *Z* = 2.215), the DEB exhibited positive spatial autocorrelation, suggesting clustering in distribution, as indicated by diffusion coefficients (S^2^/m), all exceeding 1 (Table S1). In May, high DEB areas were primarily located in the northern part of the study area. In September, high DEB areas increased towards the central, southern, and southeastern regions of the study area. In both May and September, low DEB areas were in the central–eastern region.

**Figure 6:**
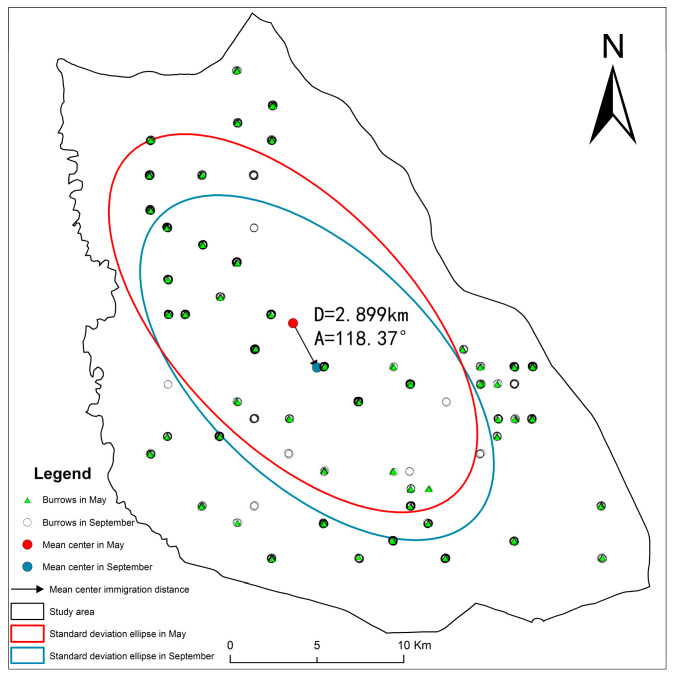
Standard deviation ellipses of burrow density in the Taiyangshan wind farm in May and September.

In May and September, the azimuth angle variation and the ellipticity change of the standard deviation ellipse were relatively small (Figure 6). This suggests a consistent and directional distribution of the DEB, predominantly following a northwest–southeast pattern, with clustering observed (Table S2). Analysing the distribution centre’s trajectory, the centre of the ellipse shifted south-eastward in September compared to May.

### 3.3. Influence of Environmental Factors

**Figure 7:**
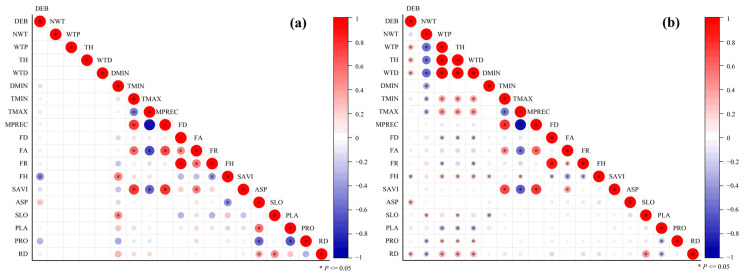
Spearman correlation coefficient between DEB and various environmental factors: (**a**) control area and (**b**) wind farm area. The abbreviations for environmental factors are as follows: NWT: the number of wind turbines within grid cells; WTP: wind turbine power; TH: tower height; WTD: wind turbine rotor diameter; DMIN: the minimum distance from the sample plot centroid to the nearest turbine; TMIN: minimum temperature; TMAX: maximum temperature; MPREC: mean precipitation; FD: flora diversity; FA: flora abundance; FR: flora richness; FH: flora height; SAVI: soil adjusted vegetation index; ASP: transformation of aspect; SLO: slope; PLA: plan curvature; PRO: profile curvature; RD: relief degree.

Spearman correlation coefficients between DEB and environmental factors are shown in Figure 7. In both the control and wind farm areas, the DEB was significantly negatively correlated with flora height (FH, *p* < 0.05). In the wind farm area (Figure 7b), the DEB showed significant positive correlations with the wind turbine power (WTP, *r* = 0.274, *p* < 0.05), tower height (TH, *r* = 0.278, *p* < 0.05), wind turbine diameter (WTD, *r* = 0.271, *p* < 0.05), transformation of aspect (ASP, *r* = 0.257, *p* < 0.05), and relief degree (RD, *r* = 0.318, *p* < 0.05). The mean flora height in the control area was significantly greater than that in the wind farm area (*p* < 0.05, Table 2), while no significant differences were observed in flora diversity or flora richness between the two areas. Flora richness and SAVI were significantly higher in September than in May, but no significant differences were found between the control and wind farm areas.

Based on collinearity tests (*r* > 0.75 and VIF > 10), wind turbine diameter, tower height, minimum temperature, and flora richness were excluded, leaving 14 environmental factors for constructing the GAM.

A GAM was applied to assess the environmental factors influencing DEB distribution within the wind farm, with the variable selection process based on the AIC, as shown in Table 3. In the control area, both flora height and profile curvature significantly influenced DEB variation (*p* < 0.05), with the DEB decreasing as these variables increased. In the wind farm area, the optimal GAM for May included wind turbine power, flora height, plan curvature, and transformation of aspect as sequential explanatory variables, collectively accounting for 36.76% of the deviance (*p* < 0.05). The optimal model for September incorporated five variables—wind turbine power, transformation of aspect, relief degree, plan curvature, and flora height—explaining 50.50% of the deviance (*p* < 0.05).

The DEB increased with higher turbine power, especially in areas with 2500 kW turbines, showing a stronger response in May than in September (Figure S1). The DEB peaked at flora heights between 10 and 20 cm and declined gradually above 20 cm. Higher DEBs were observed at plan convexity values of 110~130 and aspect values between 0.6 and 0.9 (sunny slopes). Relief degree was included only in the September model and had strong explanatory power: the DEB remained stable at relief degree values of 10~20, increased between 20 and 30, and declined above 30.

**Figure 8:**
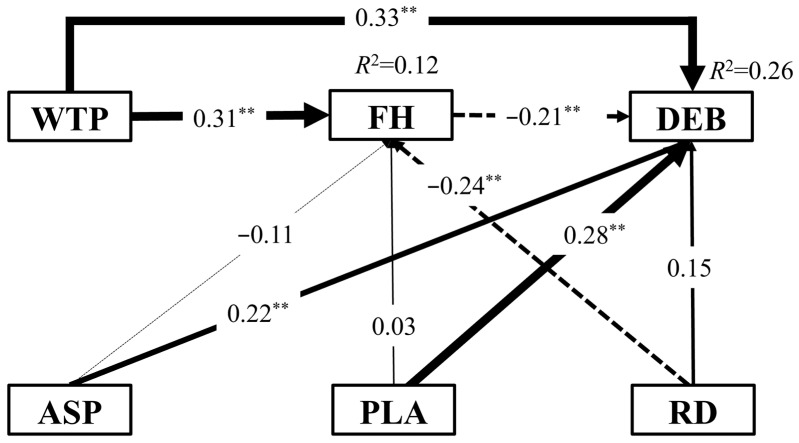
Results of the Structural Equation Model (SEM) for environmental factors influencing burrow density. Solid and dashed lines represent positive and negative effects, respectively. The width of the arrows is proportional to the strength of the effect, and the numbers on the arrows indicate standardized path coefficients. ** *p* < 0.01. The abbreviations for environmental factors are as follows: WTP: wind turbine power; FH: flora height; ASP: transformation of aspect; PLA: plan curvature; RD: relief degree.

The structural equation modelling indicated a good fit to the data (Figure 8), as evidenced by a standardized root mean square residual of 0.025 (< 0.08) and a normed fit index of 0.979 (close to 1). These values suggest a high degree of model fit. In semi-desert habitats within wind farm areas, the DEB of the Alashan ground squirrel was influenced by multiple environmental factors. Among these, wind turbine power, transformation of aspect, and plan curvature exhibited significant positive direct effects on the DEB (*p* < 0.05), with standardized path coefficients of 0.33, 0.22, and 0.28, respectively. In contrast, flora height had a significant negative direct effect (*p* < 0.05), with a coefficient of −0.21. Additionally, both wind turbine power and relief degree showed significant indirect effects on the DEB mediated through flora height (*p* < 0.05).

## 4. Discussion

### 4.1. Spatiotemporal Variation Characteristics of the Density of Burrows

Wind turbine density within wind farms is influenced by various factors, including geographical location, wind resource availability, technical specifications, and ecological constraints. Onshore wind farms typically exhibit a turbine density of 0.5~2 turbines/km^2^ [30]. However, due to the wind acceleration effects associated with mountainous terrain, the average turbine density in this study area reached 3.16 turbines/km^2^, with a maximum of 11 turbines/km^2^, indicating a highly uneven spatial distribution. Turbine densities in the 750 kW and 1500 kW power zones significantly exceeded the conventional threshold. Field surveys revealed that the highest DEB values in both May and September occurred in grids with a turbine density of approximately 2 turbines/km^2^—closely aligned with the recommended upper limit for onshore wind farms. Notably, the DEB in the 2500 kW zone was significantly higher than in all other turbine power zones and the control area (*p* < 0.05). Interestingly, the turbine density in this 2500 kW zone (1.93 turbines/km^2^) was lower than the study area’s average. This relatively sparse turbine layout helped maintain continuous herbaceous vegetation, reduced habitat fragmentation, and minimized disturbances caused by maintenance and transportation activities. These findings suggest that high-power, low-density areas within wind farms are more likely to serve as preferred habitats for Alashan ground squirrels. Furthermore, this phenomenon may be related to a reduction in predator abundance. Potential predators, such as certain raptors, might be more sensitive to high-power turbines, leading to their decreased presence. This could result in a significantly higher DEB in these high-power turbine areas compared to other regions. However, further evidence is needed to explore interspecific interactions within wind farm landscapes.

Previous studies have suggested that noise and electromagnetic radiation emitted by wind turbines may negatively impact local wildlife. Both factors exhibit distance-decay effects; for instance, low-frequency noise declines significantly beyond 200 m from turbines [24]. Within this 200 m range, soil earthworm abundance decreases notably with increasing levels of turbine-generated low-frequency noise [24]. Species such as California ground squirrels (*Otospermophilus beecheyi*) [25] and common vole (*Microtus arvalis*) [22] exhibit heightened vigilance near turbines, with the effect diminishing as distance increases. However, contrasting findings have been reported for European hamsters (*Cricetus cricetus)*, whose highest densities have been observed within 150 m of turbines—even in zones with the strongest noise and ground vibrations [27]. This may be attributed to the sound-insulating properties of their underground burrow systems. These observations suggest that the influence of turbine noise on the behaviour of soil-dwelling animals is species-specific. To avoid biases from instantaneous measurements of noise and electromagnetic radiation, this study used the distance from turbines as a proxy for assessing operational impact. Given the observed correlation between rotor diameter and noise levels in the study area [37], the rotor diameter was defined as the maximum potential disturbance radius. The results indicated that Alashan ground squirrels tend to avoid burrowing within the rotor sweep zones across different turbine power levels. Noise likely plays a key role in this avoidance behaviour, possibly due to its interference with predator detection. Additionally, frequent human activities such as turbine maintenance may further disturb Alashan ground squirrels through mechanical noise and habitat disruption.

The distribution of the DEB in the study area exhibited a pattern of multinuclear aggregation, consistent with the behavioural ecology of ground squirrels, which are gregarious-dispersive animals that generally prefer solitary living—typically occupying individual burrows with a single entrance. After leaving the maternal nest, juveniles gradually become independent, establishing burrows 20~100 m from the original nest or reusing nearby abandoned burrows [29]. The DEB values in May were slightly lower than those in September. In May, high-density areas were concentrated in the northern low-altitude region dominated by 2500 kW turbines, displaying a patchy distribution. By September, these high-density zones expanded to mid- and high-altitude areas in the central and southern parts of the study area, forming a zonal distribution. This seasonal spatial variation in DEB is likely linked to the species’ reproductive cycle: ground squirrels breed once annually, with family disintegration and juvenile dispersal occurring during summer and autumn, leading to observable shifts in population structure [29,38]. In addition, the emergence of new burrows in September may also reflect variations in breeding success, as surviving adults tend to reuse existing burrows while offspring typically disperse to establish new burrows. Despite these changes, the DEB distributions in May and September remained largely consistent, as reflected by only minor variations in the ellipticity of standard deviation ellipses. This suggests relatively stable habitat quality in the semi-desert grasslands on the study scale. Notably, the centroid of the burrow distribution ellipse shifted south-eastward from May to September, aligning with the area’s altitude and relief gradient, which increases from northwest to southeast. Post-summer, dispersing juveniles or disbanded family groups appeared to favour convex terrain, which is less susceptible to water accumulation, as a preferred site for new burrows.

### 4.2. Formation Causes Analysis of the DEB Distribution

The spatial distribution of rodents is influenced by a combination of factors, including topography, soil characteristics, vegetation structure, surface fragmentation, reproductive needs, predation pressure, and grazing intensity [29,39,40,41,42]. The ecological impacts of wind farms exhibit strong regional heterogeneity. For example, in grassland wind farms in northern China, turbine operation has been shown to elevate nighttime surface temperatures, reduce vegetation net primary productivity [12,13], and significantly degrade soil quality [14]. In contrast, wind farm operations in northwestern arid desert regions have relatively limited overall effects on vegetation, though they may locally promote shrub biomass accumulation [43]. Changes in soil and vegetation caused by wind farm operation are likely to influence the spatial distribution of soil-dwelling rodents.

This study area includes four types of turbines with varying power outputs. The effects of environmental variables on the DEB may not follow linear trends, necessitating the use of the GAM, which can capture complex nonlinear relationships through smooth functions. In this study, wind turbine power, transformation of aspect, relief degree, plan curvature, and flora height were identified as significant factors influencing the DEB. Structural equation modelling revealed that wind turbine power influenced DEB both directly and indirectly via flora height, possibly due to reduced human disturbance and distinct microclimatic conditions in high-power turbine areas. The DEB was found to significantly aggregate at vegetation heights between 10 and 20 cm and decline with increasing flora height. This preference likely reflects Alashan ground squirrels’ need for concealment and visibility for predator detection. Flora height was significantly lower in wind farm areas compared to control areas, which may be attributed to increased turbulence-induced evaporation associated with turbine operation, leading to “warming” and “drying” effects that reduce soil moisture and inhibit vegetation growth [44].

Transformation of aspect and plan curvature were positively associated with the DEB, while relief degree had an indirect effect via its influence on flora height. Alashan ground squirrels showed a preference for sunny slopes and slightly convex microtopography, less susceptible to waterlogging. Analysis showed that the DEB was higher in high-power turbine zones, where short vegetation, sunny aspects, and pronounced relief were prevalent. In contrast to European hamsters, which favour areas near turbines in farmland landscapes [27], Alashan ground squirrels tended to avoid burrowing within the rotor sweep zones of turbines. However, localized high-density burrow areas were still observed within the rotor diameter zones of 1500 kW and 2500 kW turbines, suggesting that habitat selection in Alashan ground squirrels is shaped by multiple interacting factors. Since this study was conducted during the operational phase of the wind farm, the observed patterns may also reflect behavioural adaptations, such as niche adjustment or expanded foraging ranges, allowing Alashan ground squirrels to mitigate turbine-related disturbances over time.

## 5. Conclusions

In the semi-desert steppe wind farms of northwestern China, the density of Alashan ground squirrels is the highest in areas with high-power wind turbines. The distribution of Alashan ground squirrel burrow density exhibits a certain degree of multinuclear aggregation pattern. The distribution pattern of burrows in wind farms is comprehensively influenced by wind turbine power, topographic microenvironment, and vegetation height. Alashan ground squirrels prefer to burrow in habitats featuring high-power and low-density wind farm areas, sun-facing slopes with dwarfed vegetation, convex terrain, and low-disturbance environments. Wind farm planning needs to balance turbine layout and ecological protection, reduce human disturbance, mitigate negative impacts on wild animals such as ground squirrels, and facilitate the management of grassland rodents. It is worth noting that the ecological adaptability of Alashan ground squirrels to wind farm operations still needs to be further verified through long-term monitoring and research.

## Figures and Tables

**Table 1:** Wind turbine layout of the investigated sample grids on the Taiyangshan wind farm.

Different Wind Power Zone	Number of Surveyed Grids	Number of Wind Turbines		Tower Height (m)	Wheel Diameter (m)
		Range	Average Density Mean ± SE (unit/km^2^)		
Control	14	0	0	—	—
750 kW	17	1~11	4.65 ± 0.55	50	48.4
1500 kW	13	1~6	3.69 ± 0.40	65/70/75/80	86/87/97
2000 kW	17	1~5	2.29 ± 0.34	80/85/90	110/115/120
2500 kW	14	1~3	1.93 ± 0.20	100	150

**Table 2:** Comparison of vegetation factors between wind farm and control area in May and September.

Survey Area	Survey Month	FD	FA	FR	FH	SAVI
Control area	May	0.55 ± 0.18 ns ^1^	33.03 ± 11.26 b	2.49 ± 0.56 ns	29.80 ± 18.55 a	0.17 ± 0.02 b
	September	0.62 ± 0.34 ns	65.88 ± 21.37 a	2.73 ± 1.19 ns	29.75 ± 18.77 a	0.27 ± 0.04 a
Wind farm area	May	0.62 ± 0.21 ns	31.94 ± 17.14 b	2.65 ± 0.57 ns	19.96 ± 11.18 b	0.17 ± 0.02 b
	September	0.62 ± 0.22 ns	59.30 ± 21.95 a	2.68 ± 0.64 ns	19.26 ± 10.68 b	0.26 ± 0.04 a

^1^ Different small letters mean a significant difference at the 0.05 level, and “ns” indicates no significant difference (*p* ≥ 0.05). The abbreviations for environmental factors are as follows: FD: flora diversity; FA: flora abundance; FR: flora richness; FH: flora height; SAVI: soil-adjusted vegetation index.

**Table 3:** Fitting results of the Generalized Additive Model for different wind turbine power areas.

Wind Turbine Power Area		Environmental Factor (Deviation Explanation Rate)	AIC Value	Cumulative Deviation Explanation Rate (%)	*R* ^2^	*F*	*p*
Control area		FH (26.20) + PRO (19.95) ^1^	3973.27	46.15	0.26	4.56	0.02
Wind farm area	May	WTP (14.94) + FH (8.27) + PLA (9.32) + ASP (4.23)	15,959.31	36.76	0.24	4.51	<0.001
	September	WTP (11.08) + ASP (12.89) + RD (12.64) + PLA (9.39) + FH (4.50)	9321.93	50.50	0.31	5.03	<0.001

^1^ The abbreviations for environmental factors are as follows: FH: flora height; PRO: profile curvature; WTP: wind turbine power; PLA: plan curvature; ASP: transformation of aspect; RD: relief degree.

## Data Availability

The data presented in this study are available on request from the corresponding authors.

## Supplementary Materials

The following supporting information can be downloaded at https://www.mdpi.com/article/10.3390/biology14070886/s1: Figure S1: Effect plots of significant factors in the GAM of burrow density in the Taiyangshan wind farm; Table S1: Global spatial autocorrelation parameters of the density of effective burrows in the Taiyangshan wind farm; Table S2: Shape parameters of the standard deviation ellipse for the distribution of burrows’ density in the Taiyangshan wind farm.
