# Supplementary material for: Will Wind Turbines Affect the Distribution of Alashan Ground Squirrel? Insights from Large-Scale Wind Farms in China
Source: Biology (Basel). 2025 Jul 19;14(7):886. doi: 10.3390/biology14070886 (PMC12292361; doi:10.3390/biology14070886)
Supplement: Supplementary file 1 [file biology-14-00886-s001.zip › biology-3745116-supplementary.pdf]

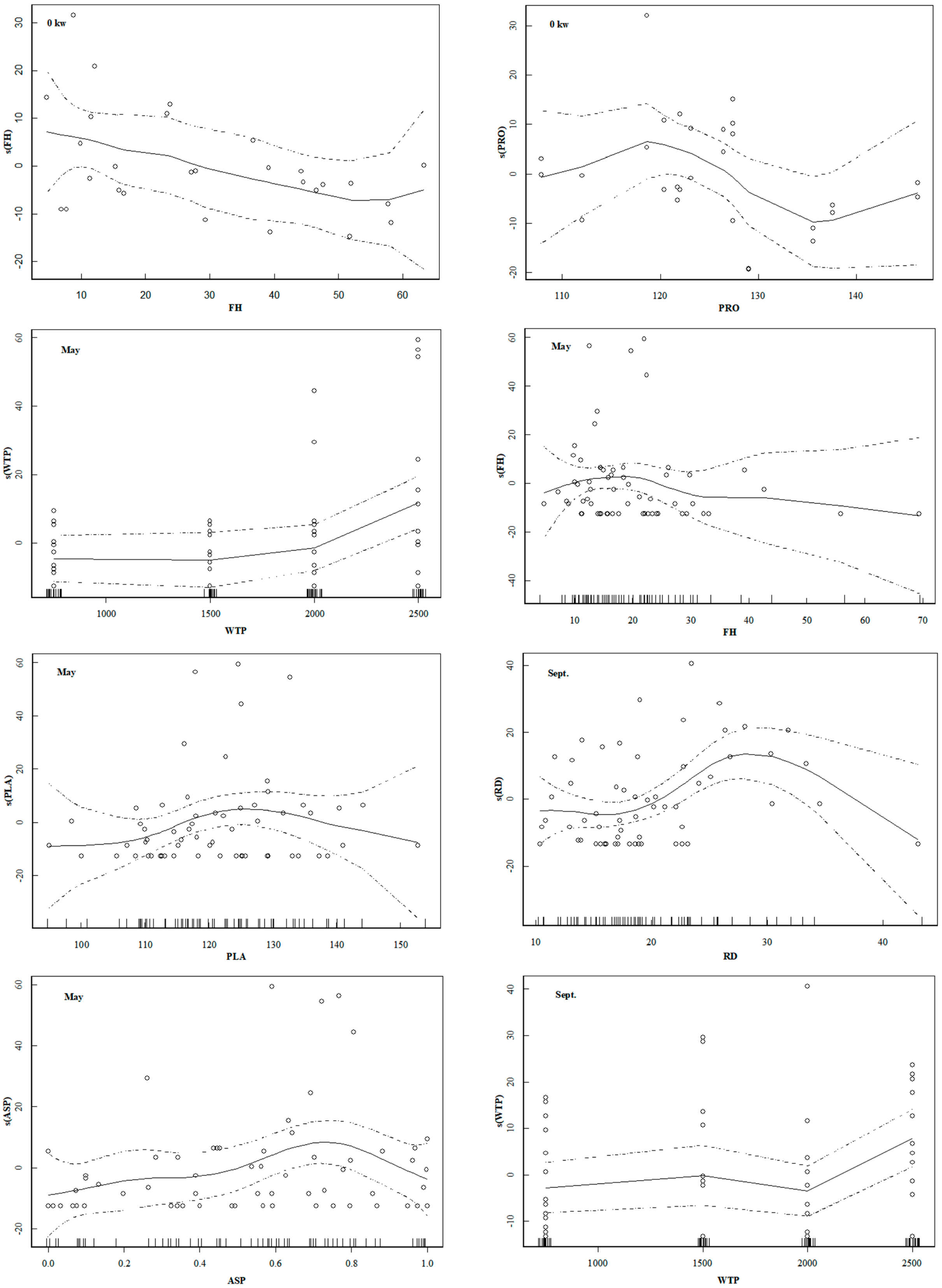
0 kw
s(FH)
FH
0 kw
s(PRO)
PRO
May
s(WTP)
WTP
May
s(FH)
FH
May
s(PLA)
PLA
Sept.
s(RD)
RD
May
s(ASP)
ASP
Sept.
s(WTP)
WTP

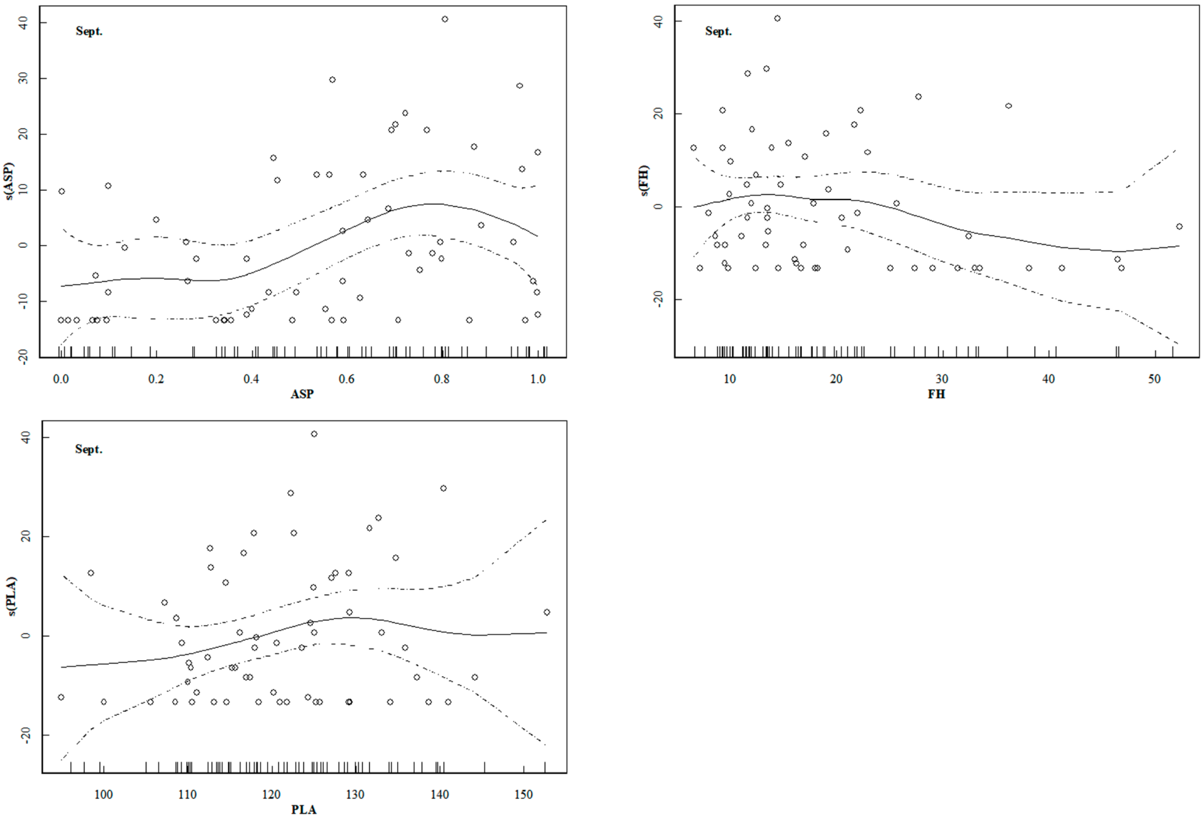

Figure S1. Effect plots of significant factors in the GAM model of burrow density in Taiyangshan wind farm.

Table S1. Global spatial autocorrelation parameters of the density of effective burrows in Taiyangshan wind farm.

Survey time	Mean value (m)	Standard deviation （δ）	Diffusion coefficient （S^2/m）	Moran's I	Z	P
May	12.87	16.28	20.60	0.151	2.647	0.006
September	1.301	13.66	14.34	0.133	2.215	0.02

Table S2. Shape parameters of the standard deviation ellipse for the distribution of burrows' density in Taiyangshan wind farm.

Survey time	Azimuth （°）	Major axis	ort axis	Oblateness
May	136.1	13.54	6.87	0.25
September	133.52	12.54	6.67	0.23
